# Correlation of peripheral blood pressure with central blood pressure and estimation of central blood pressure thresholds for diagnosis of hypertension during pregnancy

**DOI:** 10.3389/fmed.2025.1651854

**Published:** 2025-09-03

**Authors:** Xolani B. Mbongozi, Stuart D. R. Galloway, Angus Hunter, Charles B. Businge, Mirabel Nanjoh, Geoffrey A. B. Buga

**Affiliations:** ^1^Department of Obstetrics & Gynaecology, Faculty of Health Sciences, Walter Sisulu University, Mthatha, South Africa; ^2^Faculty of Health Sciences & Sport, University of Stirling, Stirling, United Kingdom; ^3^School of Science and Technology, Clifton Campus, Nottingham Trent University, Nottingham, United Kingdom; ^4^Department of Public Health, Faculty of Health Sciences, Walter Sisulu University, Mthatha, South Africa

**Keywords:** pregnant women, eclampsia, preeclampsia, hypertensive disorders of pregnancy, arterial stiffness, central and peripheral hypertension

## Abstract

**Objective:**

The main objective of the study was to evaluate the correlation and comparative use of peripheral blood pressure and central blood pressure (CBP) in identifying hypertension among pregnant women.

**Methods:**

A cross-sectional study was conducted at Nelson Mandela Academic Hospital from December 2022 to April 2024, involving 270 inpatients diagnosed with hypertensive disorders of pregnancy (HDP) and 270 normotensive controls in their second and third trimesters. Blood pressure measurements were obtained using the Microlife WatchBP Office Central, both at enrolment and within 7 days postpartum. A linear regression equation and a receiver operating characteristic curve (ROC) analysis were used to determine the thresholds for normal, mild, and severe CBP.

**Results:**

The ROC revealed that a central systolic blood pressure (cSBP) of 116 mm Hg or higher has a sensitivity of 78.5% and a specificity of 50.3% for diagnosing hypertension during pregnancy. Considering a normal peripheral diastolic pressure of less than 90 mm Hg, the upper limit of central diastolic blood pressure (cDBP) in normotensive controls was calculated to be 78 mm Hg. A significant positive correlation was found between peripheral systolic blood pressure (pSBP) and cSBP among normotensive and hypertensive women (*p* < 0.05). The median pSBP among patients with eclampsia with mild peripheral hypertension was 138.0 mmHg (IQR: 133.0–148.0) while the cSBP level in the same group was 145.0 mmHg (IQR: 140.0–150.0) mmHg. The overall median cSBP among women with eclampsia (*n* = 102) was 133.0 mmHg (IQR 120.0–143.0), and the median cDBP was 73.0 mmHg (IQR: 60.0–83.0), and these were significantly higher (*p* < 0.0001) than the median cSBP of 128.0 mmHg (IQR: 114.0–139) and cDBP of 71.0 mmHg (IQR: 61.0–81.0) among preeclamptic women.

**Conclusion:**

This study established the threshold values for central hypertension as 116/78 mmHg, with severe central hypertension defined as 148/95 mmHg or more. It reinforces the positive correlation between peripheral and CBP in both normotensive and hypertensive populations. CBP may serve as a better parameter for evaluating hypertension, especially in eclampsia. Further research is warranted to confirm these findings and to explore their clinical implications.

## Introduction

Elevated brachial blood pressure has traditionally been the main indicator for diagnosing hypertension, but with the increased availability of non-invasive central blood pressure (CBP) measuring devices, there is growing interest in using CBP for managing hypertension in non-pregnant and pregnant individuals.

The role of brachial blood pressure in the prediction of future cardiovascular structural damage, morbidity, and mortality among non-pregnant adult population is well-established in high-income countries (1), as well as in middle-income countries, such as China and Mexico (2, 3). Controlling brachial blood pressure can reduce the risk of cardiovascular death (4). However, peripheral hypertension does not necessarily correlate with maternal and perinatal outcomes, except for the increased risk of intracranial hemorrhage when there is severe peripheral hypertension (5). Numerous studies suggest that CBP is superior to peripheral blood pressure (PBP) in predicting cardiovascular disease among non-pregnant patients (6–9). The superiority of CBP is likely due to central pressures exhibiting lower variability and more accurately reflecting the tension levels on major target organs such as the heart, brain, and kidneys (10). CBP is defined as blood pressure readings taken from the aorta or common carotid arteries, primarily influenced by increased arterial stiffness and wave reflection (11). It represents the pressure against which the heart must pump to ensure blood flow throughout the body. Evidence indicates that preeclampsia (PE) is associated with increased CBP, pulse wave velocity, and arterial stiffness (12–14). It is known that the cerebrovascular complications of PE and eclampsia can occur in the presence of mild elevations of PBP and even in the absence of proteinuria (15, 16). Since the blood flow to the brain is controlled by the central aortic blood pressure, it is possible that under conditions when there is only mild elevation of the brachial blood pressure, the CBP may be much higher, thus accounting for the vasogenic oedema typical of eclampsia, justifying the need to know how the CBP behaves in patients with HDP and eclampsia.

To evaluate this hypothesis, it is essential to clearly define what constitutes mild and severe central hypertension. Current literature presents conflicting findings regarding the definition of central hypertension (17, 18). Notably, Cheng et al. have proposed a threshold CBP of 130/90 mmHg, which generally serves as a benchmark for central hypertension in non-pregnant women (11, 19). However, given the physiological changes that occur during pregnancy, it would be biased to apply findings from the non-pregnant population, especially those involving men (20). During pregnancy, physiological changes generally result in lower CBP readings compared with those in non-pregnant women and men (21). Therefore, it is necessary to establish the CBP threshold for diagnosing central hypertension in the pregnant population.

## Objectives

The primary objective of this study was to evaluate the correlation and comparative use of PBP and CBP in identifying hypertension among pregnant women. The secondary objective was to determine the threshold values for central systolic blood pressure (cSBP) and central diastolic blood pressure (cDBP) for diagnosing mild and severe central hypertension in pregnancy.

## Materials and methods

This was a cross-sectional study conducted between December 2022 and April 2024. We prospectively studied 270 inpatients diagnosed with hypertensive disorders of pregnancy (HDP) and 270 inpatient normotensive controls who were in their second and third trimesters of pregnancy. This study was conducted at the Nelson Mandela Academic Hospital, South Africa, in keeping with the principles laid down in the Helsinki Declaration (22). Walter Sisulu University and the Nelson Mandela Academic Hospital board approved this study (ethics reference number 035/2022), and informed consent was obtained from each participant before data collection.

The definitions of hypertension and HDP were based on the International Society for the Study of Hypertension in Pregnancy (23). Mild peripheral hypertension was defined as a pSBP of 110–159 mmHg and/or a pDBP of 90–109 mmHg. Severe peripheral hypertension was defined as a pSBP of 160 mmHg or higher and/or a pDBP of 110 mmHg or higher. The inclusion criteria for hypertensive patients were current hypertensive disease in pregnancy with a gestation period of ≥20 weeks. For controls, the inclusion criteria were a healthy normotensive singleton pregnancy in labor, those admitted for a repeat elective caesarean section at term, or women without hypertension presenting with particular conditions such as placenta previa, preterm premature rupture of membranes, or preterm labour. Participants were excluded if they were under 20 weeks pregnant or if they opted out of participation. Additionally, individuals with diabetes mellitus, cardiac disease, chronic renal disease, antiphospholipid syndrome, or other autoimmune diseases were also excluded from the study.

Data on the presence and type of HDP were collected from patient interviews and medical records using a standard data collection sheet. At enrolment and within 7 days after delivery, brachial and central systolic and diastolic blood pressures were measured using the Microlife WatchBP Office Central. Participants who were initially normotensive but later developed elevated blood pressure were reclassified as hypertensive, while those with gestational hypertension who later exhibited features of PE were reclassified as having PE. Before using the Microlife WatchBP Office central, the correct size of cuff was chosen and placed over the left or right upper arm so that the artery mark arrow points toward the lower arm. The cuff was wrapped around the arm, making sure that the lower edge of the cuff was approximately 2 cm above the elbow. The WatchBP Office Central device automatically measured both brachial and CBP, displaying the results on the screen once the measurement process was complete.

It is important to note that while the validity and reliability of the Microlife WatchBP Office Central have been established in the general population, its PBP measurement component lacked validation for pregnancy use (19) To address this deficiency, a pilot study was conducted involving three distinct groups of participants: 15 normotensive pregnant women, 15 women diagnosed with gestational hypertension, and 15 women with PE. Initially, PBP was measured using the pregnancy-validated Microlife WatchBP Home BT, followed by measurements with the Microlife WatchBP Office Central.

The validation process adhered to the guidelines set forth by the Association for the Advancement of Medical Instrumentation, the European Society of Hypertension, and the International Organization for Standardization (24, 25).

The results of the validation exercise demonstrated a strong correlation between the pregnancy-validated Microlife WatchBP Home BT and the Microlife WatchBP Office Central (refer to Tables 1, 2). Subsequently, the Microlife WatchBP Office Central was used to simultaneously measure both brachial blood pressure and CBP in each participant.

**Table 1:** Demographic characteristics of hypertensive cases and normotensive controls in the pilot study.

Characteristics	Hypertensive cases (*N* = 30)	Normotensive controls (*N* = 15)	Total	Student’s *t*-test *p* value
	Mean ± SD	Mean ± SD	Mean ± SD	
Age (years)	28.9 ± 7.6	24.9 ± 6.0	27.5 ± 7.3	0.082
Gravidity	2.3 ± 1.3	2.2 ± 1.7	2.3 ± 1.4	0.825
Parity	1.3 ± 0.9	1.4 ± 0.8	1.3 ± 0.9	0.715
Gestational age (wks)	34.4 ± 3.6	38.4 ± 1.5	35.7 ± 3.6	<0.0001
pSBP (mmHg)	135.7 ± 11.3	123.3 ± 10.6	131.6 ± 12.4	0.001
pDBP (mmHg)	79.7 ± 10.8	64.3 ± 13.1	74.6 ± 13.6	<0.0001
cSBP (mmHg)	130.1 ± 12.1	114.4 ± 12.5	124.9 ± 14.2	<0.0001
cDBP (mmHg)	71.6 ± 13.1	70.6 ± 11.2	71.3 ± 12.4	0.801

pSBP, peripheral systolic blood pressure; pDBP, peripheral diastolic blood pressure; cSBP, central systolic blood pressure; cDBP, central diastolic blood pressure; SD, standard deviation; wks, weeks.

**Table 2:** The absolute differences in systolic and diastolic pressures, categorized as 0–5, 0–10, and 0–15 mmHg, between the two measuring devices.

Blood pressure differences	Blood pressure categories	*n*; *N* = 45	% (95%CI)	Mean ± SD
Absolute SBP differences	0–5 mmHg	39	86.7	2.5 ± 1.5
	0–10 mmHg	44	97.8	3.0 ± 1.9
	0–15 mmHg	45	100.0	3.1 ± 2.2
Absolute DBP differences	0–5 mmHg	37	82.2	2.4 ± 1.4
	0–10 mmHg	43	95.5	3.0 ± 2.1
	0–15 mmHg	45	100.0	3.5 ± 3.1

### Statistical analysis

Data were checked for completeness and consistency before being captured using the IBM SPSS STATISTICS software package version 29 for Windows (IBM Inc., Chicago IL, USA). Two approaches were used to try to establish the upper thresholds for normal and abnormal CBP to determine what constitutes central hypertension. The first approach used a linear regression equation for calculating the cSBP and cDBP from the pSBP and cDBP of normotensive controls, and a *p*-value of <0.05 was taken as significant. The second approach used the receiver operating curve analysis for calculating the upper threshold of normal cSBP and cDBP, together with sensitivity and specificity. The Youden’s J index, sensitivity, and specificity were calculated for each blood pressure reading. Cut-off points were determined based on the index, sensitivity, specificity, and clinical relevance. The outcome variable was HDP. The lower of these thresholds from these two approaches was then chosen to represent the threshold of normal and abnormal CBP.

For the rest of the study, blood pressure readings were interpreted using medians and percentiles (p25, p75) due to the skewed nature of the data. The Kruskal–Wallis H test and the Mann–Whitney U test were employed to assess the degree of association between the continuous variables. The Wilcoxon signed-rank test was applied to compare dependent variables. A *p*-value of less than 0.05 was considered statistically significant.

## Results

### General description and socio-demographic characteristics of the sample population

**Figure 1:**
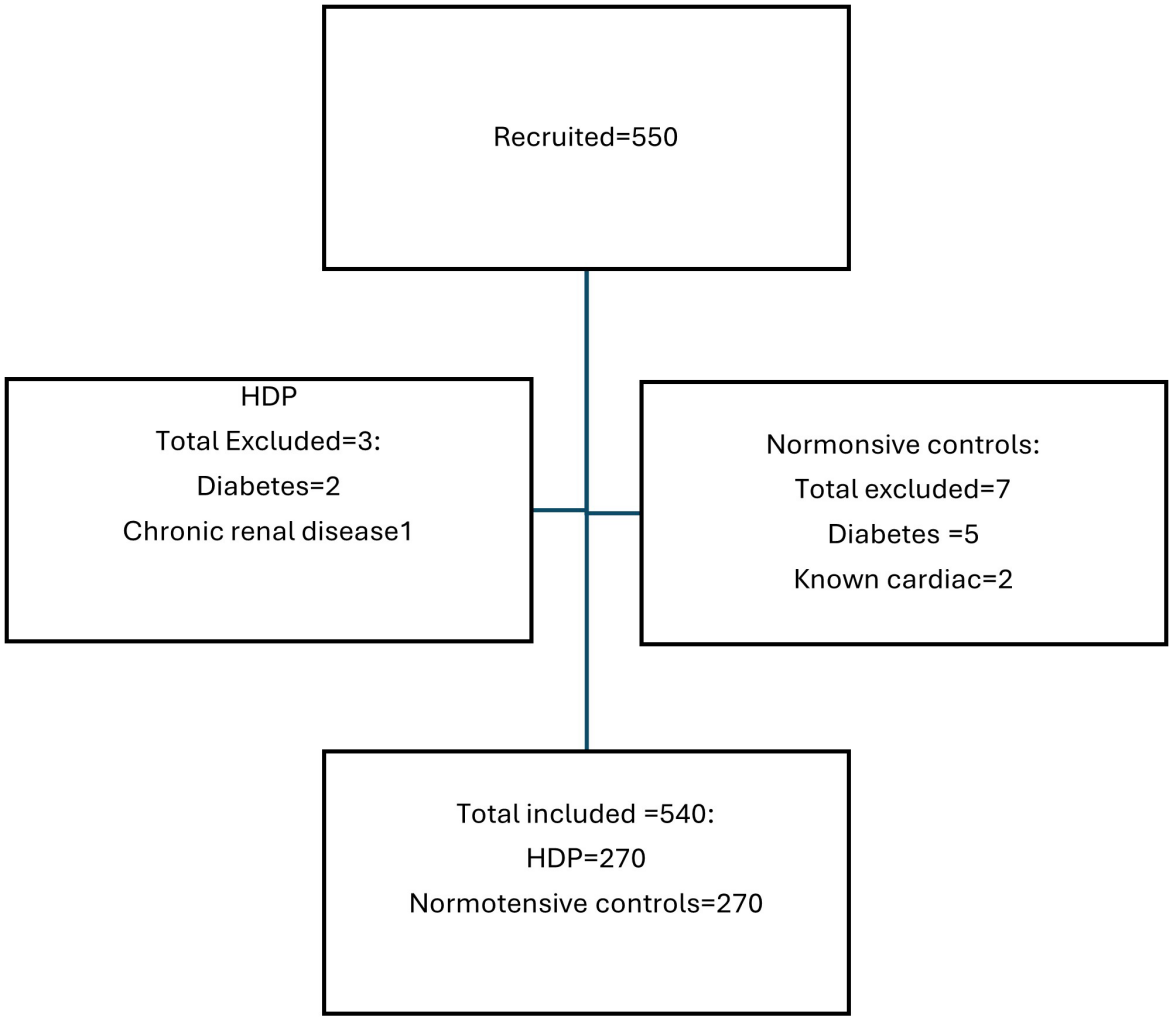
Flowchart of participant inclusion.

A total of 550 pregnant women were initially recruited for the study. However, 10 patients were excluded due to comorbidities like diabetes, chronic renal failure, and cardiac disease (Figure 1). Ultimately, 540 pregnant women were enrolled in the study, with 270 diagnosed with HDP and 270 who were normotensive controls. Of the 270 women with HDP, 143 (53%; 95% CI: 47–58.9%) had PE, 102 (37.8%; 95% CI: 32.1–43.7%) had eclampsia, 14 (5.2%; 95% CI: 3.0–8.3%) had chronic hypertension, and 11 (4.1%; 95% CI: 2.2–6.9%) had gestational hypertension. Notably, two of the eleven patients with gestational hypertension initially had normal blood pressure and were classified as controls at the time of recruitment, but subsequently developed hypertension, resulting in their reclassification. Additionally, four of the 143 preeclamptic patients initially presented with gestational hypertension but later exhibited significant proteinuria, resulting in their reclassification as PE. Of the 143 women with PE, 75 (52.4%; 95% CI: 44.3–60.5%) had PE with severe features (this number excludes women with eclampsia who form their own group) and 68 (47.6%; 95% CI: 39.5–55.7%) had PE without severe features.

The non-hypertensive controls had a median age of 28 (IQR: 24.0–33.0) years, which was significantly older than the hypertensive cases, whose median age was 25 (IQR: 20.0–32.0) years (*p* < 0.0001) (Table 3). Most participants (63.1%, *n* = 341) were aged between 20 and 34 years, while a smaller group (17.2%, *n* = 93) was under 20 years. Almost all participants (99.4%, *n* = 537) were of African descent, with the remainder being mixed race (0.6%, *n* = 3). In terms of marital status, over three-quarters (77.2%) were single and slightly more than a quarter (22.6%, *n* = 122) were married.

**Table 3:** General characteristics of hypertensives cases and normotensive controls in the main study.

Characteristics	Hypertensive cases; *n* = 270	Normotensive controls; *n* = 270	Total	Sig.**
Numerical parameters	Median (IQR)	Median (IQR)	Median (IQR)	*p*-values
Age (years)	25 (20.0–32.0)	28 (24.0–33.0)	27 (21.0–33.0)	<0.0001
Body mass index (kg/m^2)	28.5 (23.9–34.3)	29.5 (25.1–35.5)	29 (24.7–34.9)	0.071
Gestational age at recruitment	34 (31.0–37.0)	38 (36.0–39.0)	36 (33.0–38.0)	<0.0001

Categorical values	Number (percentage)	Number (percentage)	Number (percentage)	Pearson *X*^2^ Sig.
Age group (years)				
<20 years	64 (23.7)	29 (10.7)	93 (17.2)	<0.0001
20–34 years	157 (58.1)	184 (68.1)	341 (63.1)	
≥35 years	49 (18.1)	57 (21.1)	106 (19.6)	
Ethnicity				
Black African	269 (99.6)	268 (99.2)	537 (99.4)	0.624*
Mixed race	1 (0.4)	2 (0.7)	3 (0.6)	
Marital status				
Single	210 (77.8)	207 (76.7)	417 (77.2)	0.837*
Married	60 (22.2)	62 (23.0)	122 (22.6)	
Divorced	0 (0.0)	1 (0.4)	1 (0.2)	
Level of education				
Tertiary	35 (13.0)	46 (17.0)	81 (15.0)	0.194*
Secondary	207 (76.7)	189 (70.0)	396 (73.3)	
Primary	27 (10.0)	35 (13.0)	62 (11.5)	
No formal education	1 (0.4)	0 (0.0)	1 (0.2)	
Booking status				
Booked	248 (91.9)	258 (95.6)	506 (93.7)	0.111
Not booked	22 (8.1)	12 (4.4)	34 (6.3)	
Mode of delivery				
Caesarean section	233 (86.3)	235 (87.0)	468 (86.7)	0.800
Vaginal	37 (13.7)	35 (13.0)	72 (13.3)	
Underweight (BMI < 18.5)	2 (0.7)	1 (0.4)	3 (0.6)	0.196*
Normal weight (BMI 18.5–24.9)	81 (30.0)	59 (21.9)	140 (25.9)	
Overweight (BMI ≥ 25.0–29.9)	74 (27.4)	81 (30.0)	155 (28.7)	
Obese (BMI ≥ 30.0–39.9)	79 (29.3)	96 (35.6)	175 (32.4)	
Severely obese (BMI ≥ 40.0)	34 (12.6)	33 (12.2)	67 (12.4)	
HIV status				
Positive	66 (24.4)	87 (32.2)	153 (28.3)	0.056
Negative	204 (75.6)	183 (67.8)	387 (71.7)	
Viral suppression				
Unsuppressed (>1,000 cp/mL)	10 (15.2)	9 (10.3)	19 (12.4)	0.609*
Suppressed (<1,000 cp/mL)	0 (0.0)	1 (1.1)	1 (0.7)	
Less than detectable limit (<50 cp/mL)	56 (84.8)	77 (88.5)	133 (86.9)	

BMI, body mass index (kg/m^2^); cp/mL, copies/mL; *Fisher’s exact *p*-value; ** Mann–Whitney U *p*-value.

Most participants (93.7%, *n* = 434) attended antenatal clinics, while 34 (6.3%) did not. Statistically, antenatal clinic attendance was similar between the hypertensive and non-hypertensive groups (*p* > 0.05). The median gestational age at enrolment in the study was 36 (IQR: 33.0–38.0) weeks, with the non-hypertensive controls having a significantly higher median gestational age of 38 (IQR 36.0–39.0) weeks compared with the hypertensive cases at 34 weeks (IQR 31.0–37.0; *p* < 0.0001).

A significant majority of participants (86.7%, *n* = 468) delivered via caesarean section, while the remaining 13.3% (*n* = 72) had vaginal births. No significant differences in delivery methods were found between the two groups.

In terms of body weight classification, nearly one-third of the women (32.4%, *n* = 175; 95% CI = 28.6–36.4%) were classified as obese, while 12.4% (*n* = 67; 95% CI = 9.8–15.4%) were severely obese, and 0.6% (*n* = 3; 95% CI = 0.2–1.5%) were underweight.

Overall, 28.3% (*n* = 153; 95% CI = 24.7–32.2%) of the women in the study were HIV-positive, while the remainder tested negative. There was no significant difference in HIV prevalence between women with HDP and the normotensive controls (*p* > 0.05). Most of the positive for HIV women had viral loads below detectable limits, with only 12.4% (n = 19; 95% CI = 7.9–12.4%) not achieving viral suppression. Moreover, no significant differences were observed between HIV-positive women with HDP and those who were HIV-positive but normotensive regarding viral suppression (*p* > 0.05).

### Establishment of cSBP and cDBP thresholds for diagnosis of central hypertension

**Figure 2:**
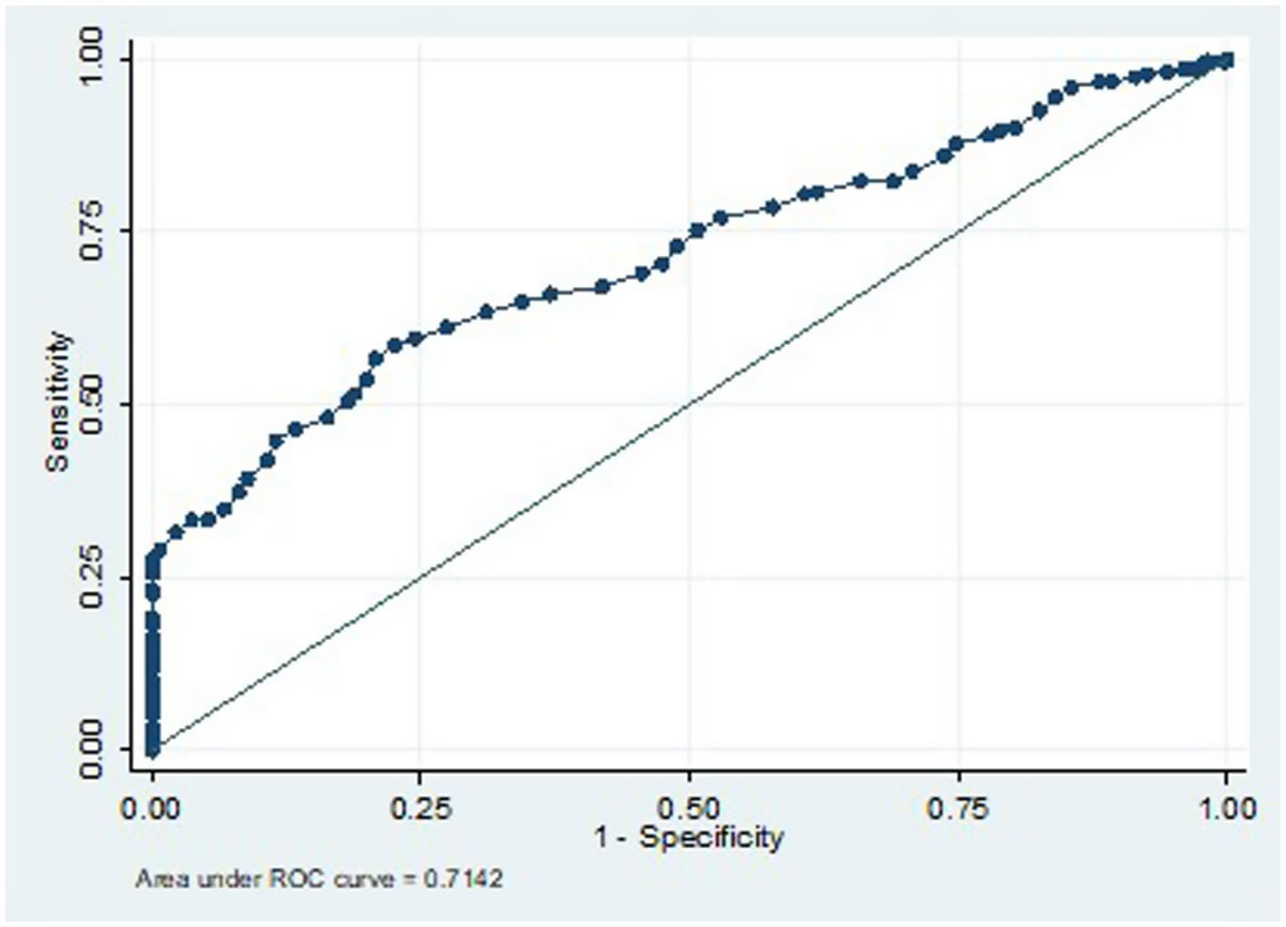
ROC for cSBP cut-off for prediction of central hypertension in women with HDP if 116 mmHg is chosen.

To establish the upper thresholds for normal and abnormal CBP, a linear regression equation and the receiver operating curve analysis were used. The lower of these thresholds was then chosen to represent the threshold of normal and abnormal CBP. Consequently, the upper threshold of normal cSBP was found to be <116 mmHg and that of the normal cDBP was <78 mmHg (Table 4 and Figure 2). CBPs above these thresholds signified central hypertension.

**Table 4:** Establishment of cSBP threshold in normotensives.

Normotensive controls (*N* = 270)		Unstandardized coefficients		Standardized coefficients	*t*	*p* value
		*B*	Std. error	Beta		
1	(Constant)	47.505	6.013		7.901	0.000
	pSBP (mmHg)	0.592	0.052	0.568	11.290	0.000

Using the simple regression formula, where predicted cSBP = a + bx (with ‘a’ representing the constant, ‘b’ as the unstandardized coefficient for pSBP, and ‘x’ as any pSBP reading), if a pSBP of 139 mm Hg is chosen as a reference point for normal systolic pressure, then the upper limit of cSBP in normotensive controls = 47.505 + 0.592 (139) = 130 mmHg.

### ROC analysis for defining cSBP thresholds for diagnosis of central hypertension during pregnancy

**Figure 3:**
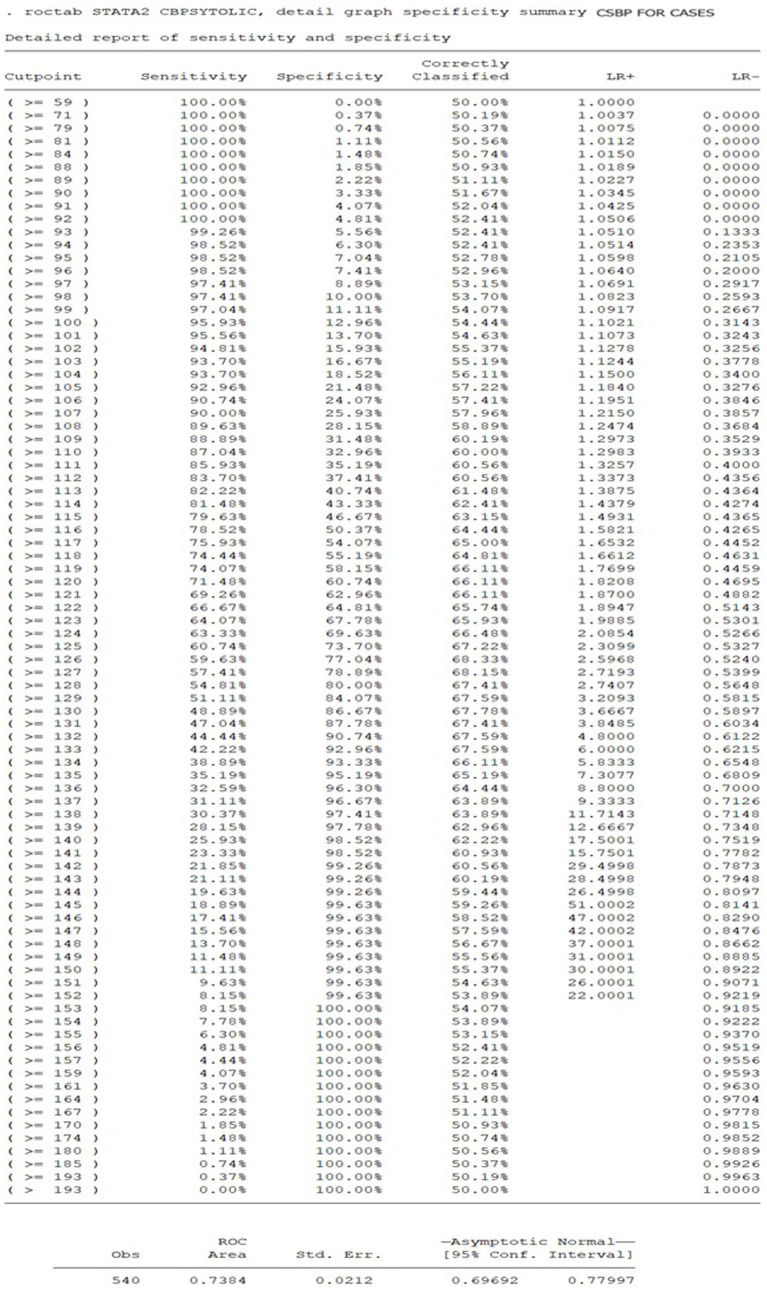
Sensitivity and specificity of cSBP cut-offs for prediction of central hypertension in women with HDP.

To predict the diagnosis of central hypertension during pregnancy, the ROC analysis results indicate that a cSBP of 116 mm Hg or higher has a sensitivity of 78.5% and a specificity of 50.3%. The cSB*p* values utilized as predictive variables for the ROC curve were derived exclusively from the HDP group. This model demonstrated a moderate discriminatory ability, with an area under the curve (AUC) of 0.7384 (Figures 2, 3).

### Linear regression equation for defining the cSBP and cDBP threshold from PBP

Based on a normal pSBP of <140 mmHg and a diastolic pressure of <90 mmHg, the upper limit of cSBP in normotensive controls was calculated to be 130 mmHg, while the upper limit of cDBP was calculated to be 78 mmHg (see Tables 4, 5) (23).

**Table 5:** Establishment of cDBP threshold in normotensives.

Normotensive (*N* = 270)		Unstandardized coefficients		Standardized coefficients	*t*	Sig.
		*B*	Std. error	Beta		
1	(Constant)	19.006	2.817		6.748	0.000
	pDBP (mmHg)	0.658	0.043	0.681	15.204	0.000

If the reference point of the pDBP in normotensive controls is taken as 89 mmHg (less than 90 mmHg), then the upper limit of cDBP in normotensive controls = 19.006 + 0.658 (89) = 77.6 or 78 mmHg.

### Establishment of thresholds for severe central hypertension

Linear regression equation was used to calculate the thresholds for severe central hypertension from the thresholds of severe PBP, defined as a pSBP of ≥160 mmHg, and/or pDBP of ≥110 mmHg, among women with HDP who were identified to have severe peripheral hypertension (23). The thresholds for severe central hypertension were found to be a cSBP of ≥148 mmHg and/or a cDBP of ≥95 mmHg (see Tables 6, 7).

**Table 6:** Establishment of cSBP thresholds for diagnosis of severe central hypertension based on pSBP of ≥160 mmHg in women with severe peripheral hypertension.

Severe peripheral hypertension		Unstandardized coefficients		Standardized coefficients	*t*	*p* value
		*B*	Std. error	Beta		
1	(Constant)	36.961	45.232		0.817	0.421
	pSBP (mmHg)	0.696	0.271	0.449	2.562	0.017

If the threshold for severe peripheral systolic blood pressure (pSBP) is taken as 160 mmHg, then the threshold for severe cSBP = 36.961 + 0.696 (160) = 148.321 or 148 mmHg.

**Table 7:** Defining cDBP cut-off for severe central hypertension based on pDBP of ≥110 mmHg in women with severe peripheral hypertension.

Severe peripheral hypertension		Unstandardized coefficients		Standardized coefficients	*t*	*p* value
		*B*	Std. error	Beta		
1	(Constant)	6.512	13.149		0.495	0.625
	pDBP (mmHg)	0.806	0.126	0.783	6.420	0.000

If the threshold for severe peripheral diastolic blood pressure (pDBP) is taken as ≥110 mmHg, then the threshold for severe cDBP = 6.512 + 0.806 (110) = 95.172 or 95 mmHg.

### Correlation of median pSBP, cSBP, pDBP, and cDBP as measured using microlife WatchBP office central among women with HDP and normotensive controls

**Figure 4:**
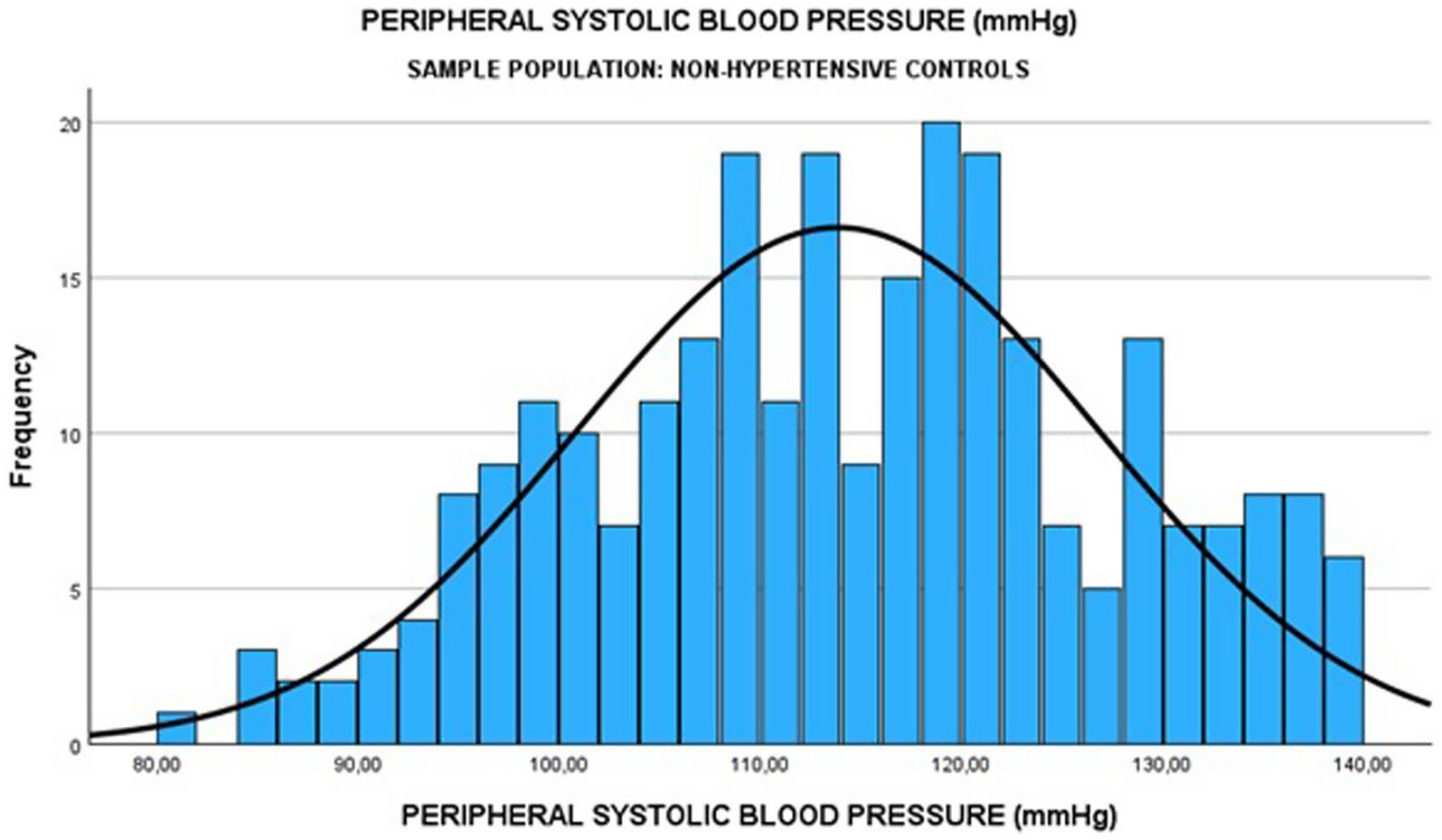
Median pSBP among normotensive pregnant women.

**Figure 5:**
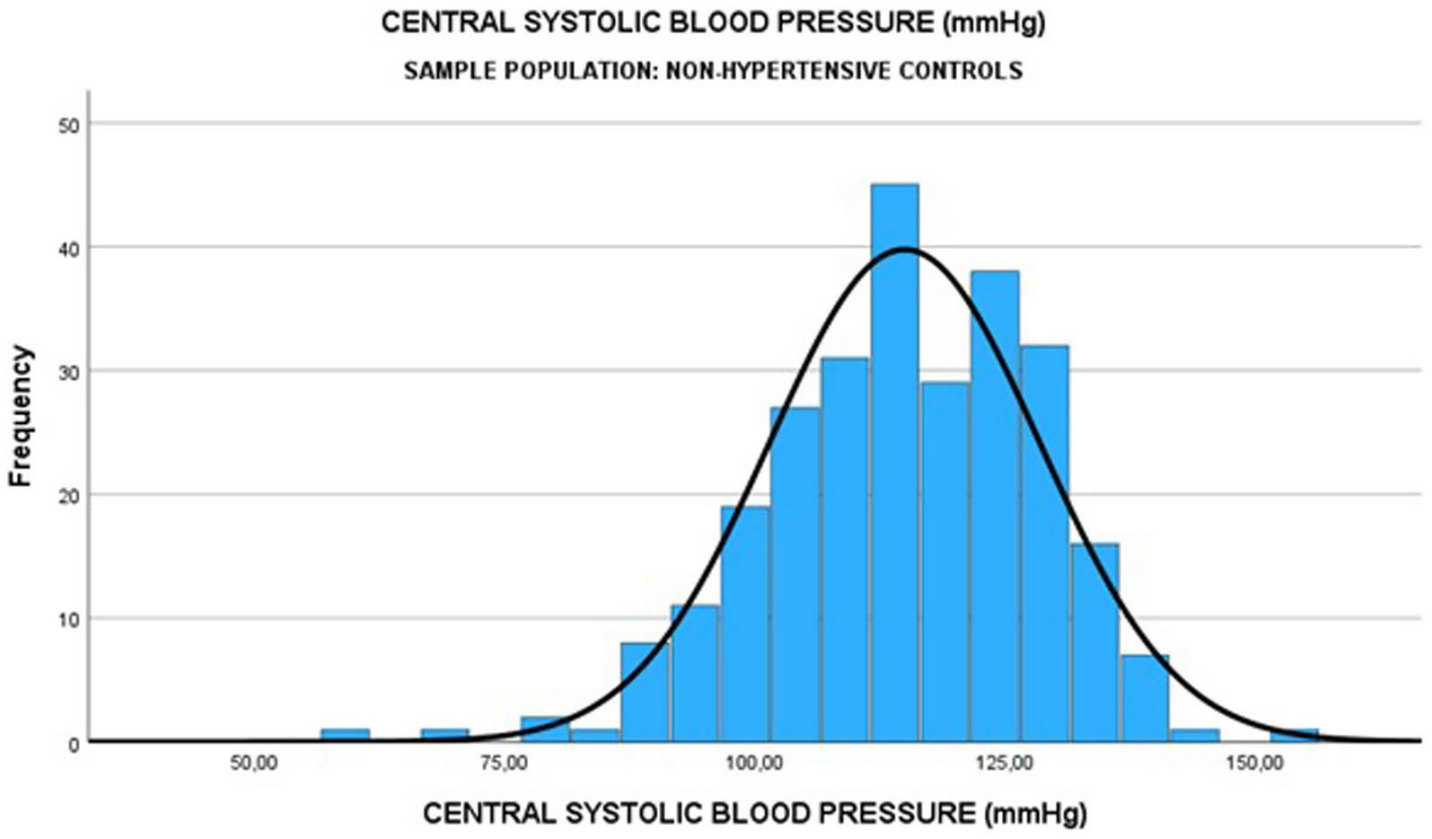
Median cSBP among normotensive pregnant women.

Among the 270 normotensive controls, the median pSBP was 114.0 (IQR: 104.0–124.0) mmHg, and the median cSBP was 115.0 (IQR: 106.0–125.0) mmHg (see Figures 4, 5). There was a strong positive relationship between the two measurements (Spearman rank correlation coefficient = 0.583; *p* < 0.0001) as shown in Table 8. The linear regression analysis revealed a positive correlation between pSBP and cSBP, with changes in pSBP being associated with a shift in the cSBP (*B* = 0.592; *p* < 0.0001). The difference between median pSBP and cSBP was 1.0 mmHg, but this was not statistically significant (Wilcoxon Signed Rank *p* = 0.122).

**Table 8:** Comparison of median pSBP and pDBP with median cSBP and cDBP among women with normal and mild peripheral hypertension as measured using Microlife WatchBP Office Central.

Hypertensive groups	cSBP median (IQR)	pSBP median (IQR)	Spearman rank	*p* value	cDBP median (IQR)	pDBP median (IQR)	Spearman rank	*p* value
Normotensive (*n* = 270)	115.0 (106.0;125.0)	114.0 (104.0;124.0)	0.583	<0.0001*	64.0 (55.0;73.0)	61.5 (52.0;69.0)	0.669	<0.0001*
Mild hypertensive (*n* = 67)	139.0 (132.0;147.0)	144.0 (138.0;148.0)	0.445	<0.0001*	82.0 (77.0;89.0)	91.0 (86.0;96.0) mmHg	0.596	<0.0001*

Normal central blood pressure = cSBP of <116 and/or cDBP <78; mild central hypertension = cSBP of 116–147 and/or cDBP of 78–94; severe central hypertension = cSBP of ≥148 and/or cDBP of ≥95 mmHg; Mild peripheral hypertension = pSBP of 140–159 and/or cDBP of 90–109. cDBP, central diastolic blood pressure; pDBP, peripheral diastolic blood pressure; cSBP, central systolic blood pressure; pSBP, peripheral systolic blood pressure. *Statistically significant.

Additionally, the median cSBP [110 (IQR: 101.0–120.0) mmHg] was less than the median pSBP [120.0 (IQR: 112.0–129.0) mmHg] in 119 participants (44%), the median cSBP [119.5 (111.0–128.0) mmHg] was greater than the median pSBP [109.0 (100.0–118.0) mmHg] in 146 participants (54%), and in 5 participants (1.9%), they were equal. The observed difference was not statistically significant (z based on positive ranks = −1.546; Wilcoxon Signed Rank *p* = 0.122).

Among the 270 normotensive controls, median cDBP was 64.0 (IQR: 55.0–73.0) mmHg, and the median pDBP was 61.5 (IQR: 52.0–69.0) mmHg. There was a strong positive correlation between the two measurements (Spearman rank correlation of 0.669, *p* < 0.0001) as shown in Table 8. Linear regression analysis also revealed that there was a positive correlation between cDBP and pDBP, with changes in pDBP being significantly associated with a shift in the cDBP (*B* = 0.658; *p* < 0.0001). The difference between median pDBP and cDBP was 2.5 mmHg, which was statistically significant (Wilcoxon Signed Rank *p* < 0.0001).

Additionally, cDBP [58.0 (50.0–66.0) mmHg] was less than pDBP [68.0 (60.0–70.0) mmHg] in 159/270 participants (59%), cDBP [64.0 (IQR: 57.0–73.0) mmHg] was greater than pDBP [57.0 (IQR: 49.0–65.0) mmHg] in 98 participants (36.3%) and was equal in only 13 participants (4.8%). The observed difference was statistically significant (*z* based on negative ranks = −4.342; Wilcoxon Signed Rank *p* < 0.0001).

### Comparison of median pSBP and median pDBP with cSBP and cDBP among women with mild peripheral hypertension as measured using microlife WatchBP office central

Among 95 women with mild peripheral hypertension, the median pSBP was 144.0(138.0–148.0) mmHg, while the median cSBP was 139.0 (132.0–147.0) mmHg as shown in Table 8. There was a strong positive relationship between the two measurements (Spearman rank *r* = 0.445; *p* < 0.0001). Linear regression analysis revealed a strong positive correlation between pSBP and cSBP, with changes in the pSBP being significantly associated with a shift in the cSBP (*B* = 0.631; *p* < 0.0001). The difference between the median pSBP and median cSBP was 1.0 mmHg, and this was statistically significant (Wilcoxon Signed Rank *p* < 0.0001).

Additionally, among the same 95 women with mild peripheral hypertension, the median cSBP was less than the median pSBP in 69 participants, and the median cSBP was greater than the median pSBP in 24 participants, while the two were equal in two participants. The analysis using the Wilcoxon Signed Rank test revealed a statistically significant difference (*p* < 0.0001).

Furthermore, among the same group of women with mild peripheral hypertension, the median pDBP was 91.0 (86.0.96.0) mmHg, while the median cDBP was 82.0 (77.0–89.0) mmHg. There was a strong positive correlation between the two measurements (Spearman’s rank *r* = 0.596; *p* < 0.0001). Linear regression analysis revealed a strongly positive correlation between the pDBP and the cDBP and cDBP, and changes in pDBP were associated with a shift in the cDBP (*B* = 0.661; *p* < 0.0001). The difference between the median pDBP and cDBP was 9.0 mmHg, which was statistically significant (*p* < 0.0001).

Additionally, among these women with mild peripheral hypertension, the median cDBP was less than the median pDBP in 81 participants, while in 14 participants, the median cDBP was greater than the median pDBP. The observed differences were statistically significant (*p* < 0.0001). Among the 14 participants with a higher median cDBP than the pDBP, four had eclampsia. The median cDBP for the four eclamptic patients was 108 mmHg, while their median pDBP was 93 mmHg. The difference between median cDBP and pDBP among the four participants was 15 mmHg, and it was statistically significant (*z* based on positive ranks = −6.5; Wilcoxon Signed Rank *p* < 0.0001).

Twenty-seven of the 102 eclamptic patients had mild peripheral hypertension, while the rest had severe peripheral hypertension. The median pSBP among eclamptic patients with mild peripheral hypertension was 138.0 mmHg (IQR: 133.0–148.0) while the cSBP level in the same group was 145.0 mmHg (IQR: 140.0–150.0).

The overall median cSBP among women with eclampsia (*n* = 102) was 133.0 mmHg (IQR 120.0–143.0) and the median cDBP was 73.0 mmHg (IQR: 60.0–83.0), and these were significantly higher (p < 0.0001) than the median cSBP of 128.0 mmHg (IQR: 114.0–139) and cDBP of 71.0 mmHg (IQR: 61.0–81.0) among preeclamptic women, with or without severe features, and much higher than the median cSBP of 115 mmHg (IQR:106.0–125.0) and cDBP of 61.5 mmHg (IQR: 52.0–69.0) among normotensive controls (Table 9).

**Table 9:** Comparison of central blood pressure readings among the different phenotypes of HDP and normotensive controls.

Blood pressure	Study groups						Kruskal-Wallis Sig.
	Control	A	B	C	D	E	
	Median (IQR)	Median (IQR)	Median (IQR)	Median (IQR)	Median (IQR)	Median (IQR)	
cSBP (mmHg)	115.0 (106.0–125.0)	132.0 (122.0–143.0)	119.5 (116.0–128.0)	128.5 (114.5–142.0)	128.0 (114.0–139.0)	133.0 (120.0–143.0)	<0.0001
cDBP (mmHg)	61.5 (52.0–69.0)	71 (63.0–80.0)	65 (61.0–71.0)	69.0 (61.0–83.0)	71.0 (61.0–81.0)	73.0 (60.0–83.0)	<0.0001

A, Gestational hypertension; B, Chronic hypertension; C, Preeclampsia without severe features; D, Preeclampsia with severe features; E, -Eclampsia; cSBP, central systolic blood pressure; cDBP, central diastolic blood pressure.

## Discussion

Historically, measuring CBP required invasive methods, which limited its application. However, recent advancements in non-invasive technologies have improved the feasibility of obtaining reliable measurements in clinical environments. This transition toward non-invasive techniques facilitates better monitoring, particularly in vulnerable groups like pregnant women.

This study aimed to assess the relationship between peripheral and CBP and to determine the thresholds for mild and severe central hypertension among pregnant women in the third trimester.

The research established that the cut-off for diagnosing central hypertension is a cSBP of 116 mmHg and a cDBP of 78 mmHg. These findings are consistent with the work of Chulkova et al. (26), who noted that a cSBP of 115 mmHg in pregnant women might indicate a risk for PE. Additionally, these results align with a study by Booysen et al. (27), which reported a cSBP upper threshold of 112 mmHg among a cohort of 311 young Black South Africans with a median age of 31. The similarity in our findings can be attributed to the younger demographic and shared background, as both studies focused on younger Black South Africans.

Conversely, research involving older populations has indicated higher thresholds for CBP. For example, a study with over 6,000 participants conducted by Hao et al. (18) established a 90th percentile cutoff for cSBP at 125 mmHg for men and 126 mmHg for women. This is consistent with previous findings from Japan, where a threshold of 129 mmHg was reported (17). Additionally, Cheng et al. (19) demonstrated through a longitudinal study involving 1,272 older participants that a central BP greater than 130/90 mmHg significantly predicts cardiovascular events over a median follow-up of 15 years. While the cut-off values determined by Cheng et al. (19) serve as important benchmarks, they may not fully apply to pregnant populations, where physiological changes result in unique cardiovascular dynamics (20). Moreover, aging is often associated with aortic stiffness, which leads to an earlier return of reflected waves in late systole, contributing to higher central BP levels (10).

For severe central hypertension, our study found that among pregnant women in their third trimester with severe brachial hypertension, cSBP was ≥148 mmHg and cDBP was ≥95 mmHg. To our knowledge, this work is the first to document the threshold for severe central hypertension. However, further studies are needed to validate these findings. These thresholds are considerably lower than those of PBP (160/110 mmHg), which is expected, as CBP is generally lower than PBP due to the inherent physiological differences between the two vascular systems.

One limitation in our calculations is that some normotensive controls displayed higher cSBP and/or cDBP values than their peripheral counterparts. Under typical conditions, cSBP should be lower than pSBP, with minimal variation between pDBP and cDBP. The possible explanation for this finding in our study is that while the participants had normal PBP, their CBP may have already been abnormal.

In comparison of peripheral and CBP, a positive correlation was found between pSBP and cSBP, as well as between pDBP and cDBP among normotensive pregnant women, aligning with previous findings by Fujime et al. (28). Importantly, Fujime et al. analyzed a larger sample of 830 participants, which adds strength to their findings. Similarly, a cross-sectional study by Priyadarsini et al. (29) involving 107 pregnant women and 53 age-matched non-pregnant women also confirmed a strong relationship between brachial systolic blood pressure and cSBP in healthy normotensive pregnant women (29).

These positive correlations could be explained physiologically by the impact of arterial elasticity on circulatory pressure distribution (30). With healthy, elastic arteries, a strong correlation between peripheral and central measurements is expected. Healthy, elastic arteries allow for a more efficient transmission of pressure waves generated by the heartbeat. When arteries are elastic, they can accommodate the surge of blood during systole, dampening the pressure peaks and maintaining a more stable blood flow throughout the body (31).

Additionally, several studies have demonstrated that CBP has a better relationship to future cardiovascular events and mortality and is also more relevant than peripheral brachial BP in predicting target organ damage, as it represents the true load imposed on the heart, brain, kidneys, and the large arteries (32–34). Nevertheless, additional research is necessary to validate these findings, given that some authors dispute the stronger correlation between CBP and cardiovascular events (35, 36).

In this study, the median cSBP among normotensive pregnant women was found to be 115 mmHg. This finding is consistent with the results of Turi et al. (37), who reported a similar mean cSBP of 115 mmHg in healthy pregnant women. However, in our study, the cSBP value was slightly higher, though not significantly, than the pSBP value of 114 mmHg that was observed. Typically, in normotensive individuals, cSBP is expected to be lower than pSBP. This atypical finding raises the possibility that individuals with higher cSBP in this study may have an underlying increase in arterial stiffness or could be at risk of developing it. As arterial stiffness increases, systolic pressure tends to rise. This is due to the early merging of the fast-reflected wave with the forward wave (38). Furthermore, increased arterial stiffness damages more elastic fibers in the blood vessel walls, leading to inadequate buffering and transmission of pulsatile pressure from the heart to target organs (39). Such changes can ultimately result in organ damage, so it is a good idea to closely monitor these patients to assess their risk of high blood pressure in the future.

In this study, cDBP in normotensive controls was less than pDBP in 159 participants, and it was greater than pDBP in 98 participants. This is again an unexpected finding in normotensive controls, as under physiological conditions, the cDBP should either be the same as the pDBP or less than the pDBP (40). This suggests that the 98 individuals with higher cDBP may be having central diastolic hypertension or are at risk of developing central hypertension, while their peripheral diastolic blood pressure puts them in the normal blood pressure category. Only a prospective follow-up study can test this explanation.

There is little correlation between the pBP and the occurrence of eclampsia. Several studies indicate that the blood pressure may be normal or only mildly elevated in women with eclampsia and even with stroke (41–43). The present study found that the median central systolic and diastolic blood pressures, as well as pDBP, were significantly higher in cases of eclampsia compared with those with other HDP and with non-hypertensive controls. This implies that CBP measurement may be better able to distinguish between the subtypes of HDP and phenotypes of PE than PBP.

In addition, the median cSBP for women with eclampsia and mild peripheral hypertension was relatively severe. This suggests that some eclamptic patients with mild pSBP actually have a cSBP that falls into the severe range. This finding may help explain why some patients with mild peripheral hypertension experience eclampsia. This implies that the cSBP might be a better predictor of eclampsia than the pSBP. Central aortic blood pressure plays a crucial role in regulating blood flow to the brain (15). Consequently, it could be inferred that in cases of eclampsia, vasogenic oedema might occur due to elevated CBP, even when brachial blood pressure remains only slightly elevated in the affected women.

The limitation of this study is that all hypertensive patients received antihypertensive treatment, which may have affected their blood pressure readings. However, patients were recruited within 24 h of admission, a time frame during which the pharmacological treatment might not have fully taken effect.

## Conclusion

This study established the threshold values for central hypertension as 116/78 mmHg, with severe central hypertension defined as 148/95 mmHg. This study also reinforces the positive correlation between peripheral and CBP in both normotensive and hypertensive populations. Notably, women with eclampsia showed higher CBP levels compared with those having mild hypertension in the severe range. This may clarify why some patients with mild peripheral hypertension experience eclampsia and may thus indicate that CBP is a better predictor of eclampsia than PBP. Further research is needed to confirm these findings.

## Data Availability

The original contributions presented in the study are included in the article/supplementary material, further inquiries can be directed to the corresponding author.
